# Short-term prediction of preeclampsia using the sFlt-1/PlGF ratio: a subanalysis of pregnant Japanese women from the PROGNOSIS Asia study

**DOI:** 10.1038/s41440-021-00629-x

**Published:** 2021-03-17

**Authors:** Akihide Ohkuchi, Shigeru Saito, Tatsuo Yamamoto, Hisanori Minakami, Hisashi Masuyama, Keiichi Kumasawa, Jun Yoshimatsu, Takeshi Nagamatsu, Angela Dietl, Sonja Grill, Martin Hund

**Affiliations:** 1 Jichi Medical University Hospital, Tochigi, Japan; 2 Toyama University Hospital, Toyama, Japan; 3 Nihon University Itabashi Hospital, Itabashi, Japan; 4 Hokkaido University, Sapporo, Hokkaido, Japan; 5 Okayama University, Okayama, Japan; 6 Osaka University, Osaka, Japan; 7 National Cerebral and Cardiovascular Center, Osaka, Japan; 8 The University of Tokyo, Tokyo, Japan; 9 Roche Diagnostics GmbH, Penzberg, Germany; 10 Roche Diagnostics International Ltd, Rotkreuz, Switzerland

**Keywords:** Japan, Predictive performance, Preeclampsia, Prognosis, Soluble fms-like tyrosine kinase 1/placental growth factor ratio

## Abstract

Two prospective multicenter studies demonstrated that a soluble fms-like tyrosine kinase 1 (sFlt-1)/placental growth factor (PlGF) ratio cutoff of ≤38 can rule out preeclampsia within 1 week with a negative predictive value (NPV) of 99.3% (PROGNOSIS) and 98.6% (PROGNOSIS Asia). We report a subanalysis of the Japanese cohort from the PROGNOSIS Asia study. Pregnant women with suspected preeclampsia between gestational weeks 18 + 0 days and 36 + 6 days were enrolled at eight Japanese sites. Primary objectives: Assess the performance of the Elecsys^®^ sFlt-1/PlGF ratio cutoff ≤38 to rule out preeclampsia within 1 week and of the cutoff >38 to rule in preeclampsia within 4 weeks. Key secondary objectives: Prediction of maternal and fetal adverse outcomes (MAOs/FAOs) and their relationship with duration of pregnancy. Of 192 women enrolled, 180 (93.8%)/175 (91.1%) were evaluable for primary/combined endpoint analyses. Overall preeclampsia prevalence was 13.3%. A sFlt-1/PlGF ratio of ≤38 provided an NPV of 100% (95% confidence interval [CI], 97.5–100) for ruling out preeclampsia within 1 week, and a ratio of >38 provided a positive predictive value of 32.4% (95% CI, 18.0–49.8) for ruling in preeclampsia within 4 weeks. The area under the curve for the prediction of preeclampsia/maternal/fetal adverse outcomes within 1 week was 94.2% (95% CI, 89.3–97.8). After adjusting for gestational age and final preeclampsia status, Cox regression indicated a 2.8-fold greater risk of imminent delivery for women with a sFlt-1/PlGF ratio >38 versus ≤38. This subanalysis of Japanese women with suspicion of preeclampsia showed high predictive value for a Elecsys sFlt-1/PlGF ratio cutoff of 38 for short-term prediction of preeclampsia.

## Introduction

Preeclampsia is a heterogeneous, multiorgan disorder affecting 2–5% of pregnancies worldwide and at least 2.7% of singleton pregnancies in Japan [1–6]. The condition is associated with significant maternal and fetal mortality, with hypertensive disorders of pregnancy accounting for ~14% of maternal deaths globally [7].

Triage of women presenting with clinically suspected preeclampsia is challenging, and effective care requires identification and referral of women at high risk [8, 9]. The current “gold standard” for the diagnosis of preeclampsia is based on the presence of new-onset hypertension plus proteinuria and/or other maternal organ dysfunction [1, 10, 11]. However, the predictive value of blood pressure and other clinical characteristics for preeclampsia or adverse pregnancy outcomes is relatively low [12, 13]. Poor prediction of preeclampsia may lead to unnecessary hospitalization of women who will not develop the condition, while others who develop preeclampsia may be overlooked. Therefore, an improved method for the prediction of preeclampsia and associated adverse maternal/fetal outcomes is required.

Soluble fms-like tyrosine kinase 1 (sFlt-1), placental growth factor (PlGF), and vascular endothelial growth factor (VEGF) have been shown to play a pathogenic role in the development of preeclampsia [14]. sFlt-1, an antagonist of PlGF and VEGF, is upregulated in the preeclamptic placenta, leading to increased systemic levels of sFlt-1 and resultant decreases in the circulating levels of PlGF and VEGF [14]. The sFlt-1/PlGF ratio has been shown to be elevated in pregnant women 4–5 weeks prior to clinical onset of preeclampsia [15, 16] and is a reliable tool for discriminating between different types of pregnancy-related hypertensive disorders and for predicting imminent delivery [17, 18]. Two prospective multicenter studies have demonstrated that a sFlt-1/PlGF ratio cutoff of ≤38 can rule out preeclampsia within 1 week with a negative predictive value (NPV) of 99.3% (PROGNOSIS) and 98.6% (PROGNOSIS Asia) [19, 20]. Here, we report the findings from an exploratory subanalysis of the PROGNOSIS Asia study to examine the performance of the sFlt-1/PlGF ratio for the short-term prediction of preeclampsia in the Japanese cohort.

## Methods

### Study design

PROGNOSIS Asia was a prospective, blinded, multicenter, and observational study conducted across 25 sites in Asia between December 2014 and December 2016; the results of the primary analysis have been reported previously [20].

The Japanese cohort was enrolled at eight sites in Japan (June 2015–May 2016). Diagnostic criteria were based on the International Society for the Study of Hypertension in Pregnancy guidelines [21]. Key inclusion criteria were pregnant women ≥18 years of age at gestational week 18 + 0 days to 36 + 6 days presenting with suspected preeclampsia per protocol-defined criteria, previously published by Bian et al. [20]. Inclusion criteria were aligned with local practice guidelines, whereby the criterion relating to the lower limit of blood pressure was adapted to reflect Japanese practice and gestational week was adapted to 18 weeks (rather than 20 weeks of gestation for the other countries). Key exclusion criteria were manifest preeclampsia or confirmed diagnosis of hemolysis, elevated liver enzymes, low platelet count (HELLP) syndrome, multiple pregnancy, confirmed diagnosis of a fetal chromosomal abnormality, or having received treatment with an investigational medicine within 90 days.

The protocol was approved by local ethics committees and institutional review boards at each of the eight sites prior to study initiation. All participants provided written informed consent, and the study was conducted in accordance with the principles of the Declaration of Helsinki and International Conference on Harmonization guidelines for Good Clinical Practice.

### Study assessments

Assessments were made at visit 1 (baseline); visit 2 (7–14 days from baseline); visit 3 (24–32 days from baseline); at delivery; and at the postpartum visit. Unscheduled visits occurred in the event of pregnancy complications. Clinical data (medical history, clinical assessments) were collected at all study visits. Serum samples were collected at visits 1–3 according to a standardized operating procedure and stored frozen at −70 °C or −80 °C. Samples were analyzed at a College of American Pathologists-accredited central laboratory in Singapore (Covance Central Laboratory Service, Singapore), where maternal serum levels of sFlt-1 and PlGF were determined by fully automated Elecsys^®^ sFlt-1 and PlGF immunoassays on a Cobas e 601 analyzer (Roche Diagnostics, Mannheim, Germany) [22]. The sFlt-1/PlGF ratio for each sample was calculated by the Roche biostatistics department (Penzberg, Germany), following transfer of all assay results at the end of the study.

### Analysis objectives

The primary objectives were to validate the Elecsys sFlt-1/PlGF ratio cutoff of ≤38 to predict the absence of preeclampsia/eclampsia/HELLP syndrome within 1 week of the baseline visit and to validate the Elecsys sFlt-1/PlGF ratio cutoff of >38 to predict the occurrence of preeclampsia/eclampsia/HELLP syndrome within 4 weeks of the baseline visit. Key secondary objectives were to investigate the value of the sFlt-1/PlGF ratio for predicting maternal and fetal adverse outcomes (MAOs, FAOs) within 1 week or 4 weeks and to investigate the relationship between a sFlt-1/PlGF ratio of >38 and time to delivery and preterm delivery. The associations between the sFlt-1/PlGF ratio and the combined endpoint of preeclampsia and/or MAO and/or FAO were also examined. MAOs were defined as any preeclampsia-related adverse outcome other than preeclampsia/eclampsia/HELLP syndrome (e.g., maternal death, pulmonary edema, acute renal failure, cerebral hemorrhage); FAOs included perinatal/fetal death, delivery <34 weeks, fetal growth restriction, placental abruption, neonatal respiratory distress syndrome, necrotizing enterocolitis, and intraventricular hemorrhage.

### Statistical analyses

Sample size was calculated for the entire study population of PROGNOSIS Asia [20], not for analyses in subset cohorts. The enrollment target for Japan was a minimum of 145 individuals.

Analyses were conducted using SAS version 9.4 (SAS, Cary, NC, USA) and R version 3.2.2 and version 3.4.0 (R Foundation, Vienna, Austria). Descriptive statistics were reported as medians and interquartile ranges for continuous data and as absolute and relative frequencies for count data. The predictive performance of the sFlt-1/PlGF ratio was determined for each objective by estimation of the NPV, positive predictive value (PPV), sensitivity, specificity, and area under the receiver-operator characteristic (ROC) curve, each with corresponding 95% confidence intervals (CI). A *p* value of < 0.05 defined a statistically significant difference. Cox regression was used to determine the impact of the sFlt-1/PlGF ratio on remaining pregnancy duration at the time of blood sampling, dichotomized by sFlt-1/PlGF ratios (≤38 vs. >38) and adjusted for gestational age and final preeclampsia status.

## Results

### Analysis population

**Fig. 1:**
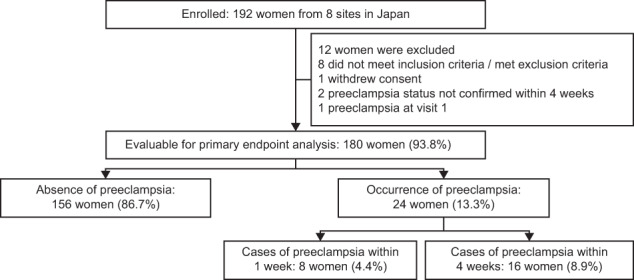
Participant disposition

A total of 192 women were enrolled at eight sites in Japan; 180 (93.8%) were evaluable and included in the primary objective analyses (Fig. 1). The overall prevalence of preeclampsia was 13.3% (4.4% within 1 week; 8.9% within 4 weeks; Fig. 1), and the most common reasons for suspected preeclampsia were new onset of elevated blood pressure (48.3%), abnormal uterine perfusion (32.8%), and new onset of protein in the urine (31.1%; Table 1).

**Table 1:** Baseline participant demographics and characteristics for the Japanese cohort of the PROGNOSIS Asia study, for all participants and according to preeclampsia status

Characteristic^a, b^	All participants (*N* = 180)	No preeclampsia at any time (*n* = 156)	Preeclampsia at any time (*n* = 24)
Age, years	35.0 (31.5–38.0)	35.0 (31.0–38.0)	35.0 (32.0–39.0)
Gestational age at baseline, weeks	31.7 (27.4–34.6)	31.6 (27.3–34.6)	32.2 (28.2–35.1)
Gestational age at delivery, weeks	38.1 (37.1–39.3)^c^	38.3 (37.4–39.4)^c^	36.3 (34.1–38.5)
Blood pressure at baseline, mmHg			
Systolic	128.0 (111.0–136.0)	125.0 (109.0–134.0)	138.0 (132.0–147.0)
Diastolic	75.0 (64.0–83.0)	72.0 (61.5–82.0)	84.0 (80.0–94.5)
Prepregnancy BMI, kg/m^2^	21.6 (20.1–24.3)^d^	21.5 (20.0–24.0)^e^	22.2 (20.1–25.3)^c^
Reasons for suspected preeclampsia, *n* (%)^f^			
New onset of elevated blood pressure	87 (48.3)	72 (46.2)	15 (62.5)
Aggravation of preexisting hypertension	14 (7.8)	9 (5.8)	5 (20.8)
New onset of protein in urine	56 (31.1)	45 (28.8)	11 (45.8)
Aggravation of existing proteinuria	0	0	0
Epigastric pain	0	0	0
Visual disturbances	2 (1.1)	1 (0.6)	1 (4.2)
Abnormal uterine perfusion	59 (32.8)	51 (32.7)	8 (33.3)
Partial HELLP syndrome	5 (2.8)	4 (2.6)	1 (4.2)
Height of neonate, cm	47.5 (45.2–50.0)^d^	48.0 (45.6–50.0)^d^	46.1 (42.5–48.1)
Weight of neonate, g	2705 (2272–3146)^c^	2796 (2348–3214)^c^	2286 (1644–2796)

*BMI* body mass index, *HELLP* haemolysis, elevated liver enzymes, low platelet count

^a^Data are reported as median (interquartile range) unless stated otherwise

^b^Statistically significant differences were observed between women who developed preeclampsia and women who did not develop preeclampsia for the following characteristics: median blood pressure at baseline (*p* < 0.001), median gestational age at delivery (*p* < 0.001), and median height (*p*  = 0.019) and median weight of the neonate (*p*  = 0.002). For median gestational age at baseline, *p* = 0.429

^c^Missing information for one participant

^d^Missing information for three participants

^e^Missing information for two participants

^f^Some women had more than one reason for suspected preeclampsia

The characteristics of women who developed preeclampsia at any time and those who did not develop preeclampsia at any time are shown in Table 1. Median age and prepregnancy body mass index at baseline were comparable between women who did and did not develop preeclampsia. Median blood pressure was higher in women who developed preeclampsia than in those who did not develop preeclampsia (*p* < 0.001), whereas the median gestational age at delivery (*p* < 0.001) and median height and weight of the neonate (*p* = 0.019 and *p* = 0.002, respectively) were lower in women who developed preeclampsia than in those who did not develop preeclampsia.

### Prediction of preeclampsia

**Fig. 2:**
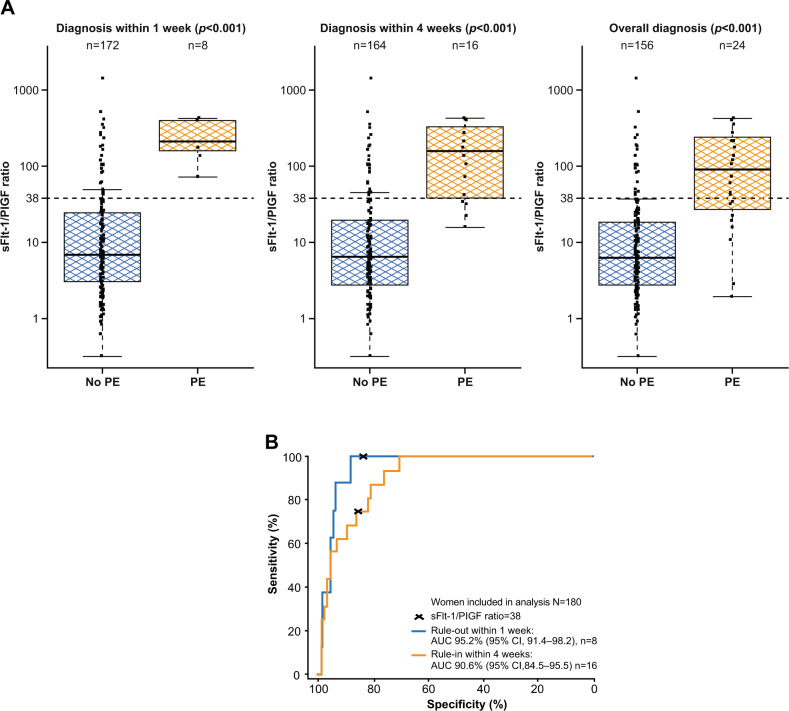
Performance of the sFlt-1/PlGF ratio for predicting preeclampsia within 1 week and within 4 weeks. **A** Shows the distribution of sFlt-1/PlGF ratios at baseline and *p* values for participants who developed or did not develop preeclampsia within 1 week, within 4 weeks and overall.^a^ **B** Shows the performance of the ratio for ruling out preeclampsia within 1 week (blue) and ruling in preeclampsia within 4 weeks (orange). ^a^Boxes represent the median and interquartile range; the lower whisker represents the larger of the minimum ratios and the 25th quartile to 1.5× interquartile range, while the higher whisker represents the smaller of the maximum ratios and the 75th quartile to 1.5× interquartile range, in log-scale. AUC area under the curve, CI confidence interval, PE preeclampsia, PlGF placental growth factor, sFlt-1 soluble fms-like tyrosine kinase 1

Median sFlt-1/PlGF ratio values were higher in women who developed preeclampsia than in those who did not develop preeclampsia (Fig. 2A after 1 week (212.4 vs. 6.8; *p* < 0.001), 4 weeks (157.7 vs. 6.4; *p* < 0.001) and overall (88.8 vs. 6.4; *p* < 0.001).

The sFlt-1/PlGF ratio showed high sensitivity and specificity for ruling out preeclampsia within 1 week (ratio ≤38) and ruling in preeclampsia within 4 weeks (ratio >38) (Table 2). Based on a sFlt-1/PlGF ratio ≤38, the NPV for ruling out preeclampsia within 1 week was 100% (95% CI, 97.5–100.0), and the corresponding area under the ROC curve was 95.2% (95% CI, 91.4–98.2) (Fig. 2B). Based on a sFlt-1/PlGF ratio >38, the PPV for ruling in preeclampsia within 4 weeks was 32.4% (95% CI, 18.0–49.8), and the corresponding area under the ROC curve was 90.6% (95% CI, 84.5–95.5) (Fig. 2B).

**Table 2:** Performance of the sFlt-1/PlGF ratio using a cutoff of 38 for short-term prediction of preeclampsia

Preeclampsia	TN/FN	TP/FP	NPV, % (95% CI)	PPV, % (95% CI)	Sensitivity, % (95% CI)	Specificity, % (95% CI)
Within 1 week	143/0	8/29	**100.0 (97.5–100.0)**^**a**^	21.6 (9.8–38.2)	100 (63.1–100.0)	83.1 (76.7–88.4)
Within 4 weeks	139/4	12/25	97.2 (93.0–99.2)	**32.4 (18.0–49.8)**^**a**^	75.0 (47.6–92.7)	84.8 (78.3–89.9)

Bold entries indicate significant NPV/PPV values.

*CI* confidence interval, *FN* false negative, *FP* false positive, *NPV* negative predictive value, *PlGF* placental growth factor, *PPV* positive predictive value, *sFlt-1* soluble fms-like tyrosine kinase 1, *TN* true negative, *TP* true positive

^a^Primary study objective; for rule out of preeclampsia within 1 week, an sFlt-1/PlGF ratio of ≤38 was used. For rule in of preeclampsia within 4 weeks, an sFlt-1/PlGF ratio of >38 was used

### Prediction of adverse outcomes

A total of 179 participants were eligible for analysis of MAOs. The predictive performance of the sFlt-1/PlGF ratio for MAOs could not be assessed, as only one participant experienced one or more MAOs (central serous chorioretinopathy, pulmonary edema, acute renal failure, and disseminated intravascular coagulation); the participant developed preeclampsia after 4 weeks and had a sFlt-1/PlGF ratio at baseline of 349.8.

A total of 176 participants were eligible for analysis of FAOs. One or more FAOs occurred in 6 individuals within 1 week and in 36 individuals within 4 weeks; at both timepoints, median sFlt-1/PlGF ratios were higher in participants with versus without an FAO (Table 3). Within participants with one or more FAOs, sFlt-1/PlGF ratios were higher in women ruled in for preeclampsia within 1 week and within 4 weeks than in women who were ruled out. The area under the ROC curve for any FAO within 4 weeks in all participants was 82.6% (95% CI, 75.0–89.5) (Table 3; Supplementary Fig. 1).

**Table 3:** Distribution of sFlt-1/PlGF ratios by FAO status within 1 week and within 4 weeks, in all women and by preeclampsia status^a^

FAO status (without/with preeclampsia^b^)	*N*	sFlt-1/PlGF ratio, median (IQR)
FAO within 1 week	6	312.3 (71.4–397.6)
Women without preeclampsia within 1 week	2	134.1 (36.9–231.4)
Women with preeclampsia within 1 week	4	395.4 (232.3–412.2)
No FAO within 1 week	170	6.8 (2.9–26.1)
Women without preeclampsia within 1 week	166	6.6 (2.8–23.6)
Women with preeclampsia within 1 week	4	193.8 (157.7–212.4)
FAO within 4 weeks	36	66.1 (11.6–242.0)
Women without preeclampsia within 4 weeks	28	25.7 (9.3–120.8)
Women with preeclampsia within 4 weeks	8	330.8 (141.8–405.1)
No FAO within 4 weeks	140	5.4 (2.5–18.2)
Women without preeclampsia within 4 weeks	133	5.0 (2.4–12.0)
Women with preeclampsia within 4 weeks	7	41.7 (31.3–210.1)

*FAO* foetal adverse outcome, *HELLP* haemolysis, elevated liver enzymes, low platelet count, *IQR* interquartile range, *PlGF* placental growth factor, *sFlt-1* soluble fms-like tyrosine kinase 1

^a^176 participants from Japan were eligible for this analysis

^b^Preeclampsia/eclampsia/HELLP syndrome

**Fig. 3:**
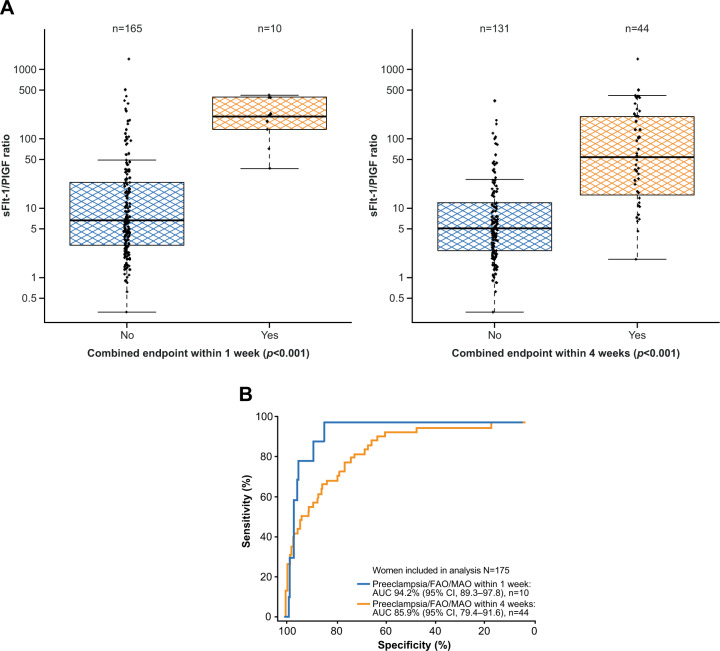
Performance of the sFlt-1/PlGF ratio for predicting preeclampsia/MAO/FAO within 1 week and within 4 weeks.^a^ **A** shows the distribution of sFlt-1/PlGF ratios at baseline and *p* values for participants who developed or did not develop preeclampsia/MAO/FAO within 1 week and within 4 weeks.^b^ **B** shows the performance of the ratio for predicting preeclampsia/MAO/FAO within 1 week (blue) and within 4 weeks (orange). ^a^175 participants from Japan were eligible for this analysis. ^b^Boxes represent the median and interquartile range; the lower whisker represents the larger of the minimum ratios and the 25th quartile to 1.5× interquartile range, while the higher whisker represents the smaller of the maximum ratios and the 75th quartile to 1.5× interquartile range, in log-scale. AUC area under the curve, CI confidence interval, FAO fetal adverse outcome, MAO maternal adverse outcome, PlGF placental growth factor, sFlt-1 soluble fms-like tyrosine kinase 1

In total, 175 participants were eligible for analysis of the combined endpoint. In these individuals, a sFlt-1/PlGF ratio of 38 provided good separation of women with and without the combined endpoint of preeclampsia/eclampsia/HELLP syndrome and/or MAO and/or FAO within 1 or 4 weeks (both *p* < 0.001; Fig. 3A). The area under the ROC curve (95% CI) for predicting the combined endpoint within 1 week was 94.2% (89.3–97.8), and within 4 weeks, it was 85.9% (79.4–91.6) (Fig. 3B).

### Correlation between sFlt-1/PlGF and delivery

Based on data for 179 eligible participants, a sFlt-1/PlGF ratio >38 at baseline was associated with a shorter pregnancy duration on average, regardless of preeclampsia development (Supplementary Fig. 2). Cox regression analyses showed that the estimated likelihood of imminent delivery was 2.8-fold (95% CI, 1.8–4.2) higher in women with a sFlt-1/PlGF ratio >38 versus a ratio ≤38 after adjustment for gestational age and preeclampsia status. In women who developed preeclampsia, median gestational age did not reach that of full term (≥37 weeks), and among women who did not develop preeclampsia (*n* = 139), median sFlt-1/PlGF ratios were numerically higher in women with preterm deliveries (<37 weeks, either initiated by a physician [iatrogenic] or not [noniatrogenic]) than in women who delivered at term (≥37 weeks) (Supplementary Fig. 3).

## Discussion

The present subanalysis validates the sFlt-1/PlGF ratio cutoff of 38 for short-term prediction of preeclampsia, maternal/fetal adverse outcomes and preterm delivery in Japanese women with clinically suspected preeclampsia. Importantly, a sFlt-1/PlGF ratio cutoff of ≤38 provided an NPV of 100% (95% CI, 97.5–100.0). This will enable clinicians in Japan to rule out preeclampsia within 1 week with a high degree of confidence, thus informing their decision making regarding hospitalization or outpatient monitoring. Additionally, a sFlt-1/PlGF ratio cutoff of >38 provided a PPV of 32.4% (95% CI, 18.0–49.8) to rule in preeclampsia within 4 weeks, which is higher than previously reported PPVs for predictors such as antepartum and intrapartum blood pressures combined (18–20%) and comparable to PPVs based on antepartum blood pressure only (22–36%) [12].

With the exception of slight differences in the inclusion criteria for the Japanese cohort (in line with local practice guidelines), the eligibility criteria in the Japanese and overall PROGNOSIS Asia study cohorts were well matched, and the same diagnostic criteria were applied to the entire population, permitting direct comparison [20]. In this regard, baseline characteristics in the Japanese cohort were generally similar to those for the overall population, including a similar prevalence of preeclampsia (13.3% versus 14.4%, respectively). Key measures of the predictive performance of the sFlt-1/PlGF ratio were also consistent in the Japanese and overall study cohorts, including the NPV for ruling out preeclampsia within 1 week (100% versus 98.6%, respectively), PPV for ruling in preeclampsia within 4 weeks (32.4% versus 30.3%), area under the ROC curve for any FAO within 4 weeks (82.6% versus 83.1%) and risk of imminent delivery in women with sFlt-1/PlGF ratio >38 versus ≤38 (2.8-fold higher versus 3.5-fold higher); for each of these measures, the 95% confidence intervals overlapped between cohorts [20].

Our results were also consistent with those of the PROGNOSIS study, which enrolled a predominantly Caucasian population and reported an incidence of preeclampsia and/or HELLP syndrome of 17.8% in the validation cohort [19]. The baseline characteristics of the Japanese cohort were similar to those of the PROGNOSIS validation cohort, although there were some differences. In the Japanese cohort, the median age (35 years versus 32 years) was higher than in the PROGNOSIS validation cohort of patients from non-Asian countries [19]. This is perhaps to be expected; advanced maternal age (AMA) (≥35 years at the birth of one’s first child) has become increasingly common in Japan, rising from a rate of 8.6% in 1990 to 25.9% in 2012 [23]. Among women of AMA, such as those within the Japanese cohort, there is some discourse surrounding levels of sFlt-1, which should be taken into consideration; it was recently reported that in AMA murine models resembling human AMA, serum sFlt-1 levels were significantly lower than in controls (*p* < 0.05), suggesting serum sFlt-1 levels are not necessarily reflective of preeclampsia pathogenesis in this setting [24]. This may have implications for the use of the sFlt-1/PlGF ratio in AMA women; however, further investigation in a human population is required. Other differences between the cohorts include the lower median BMI (22.2 kg/m^2^ versus 26.4 kg/m^2^) and lower rate of preeclampsia (13.3% versus 17.8%) noted in the Japanese cohort compared with the PROGNOSIS validation cohort [19]. However, the sFlt-1/PlGF ratio still demonstrated a comparable predictive performance between cohorts in terms of the NPV for ruling out preeclampsia within 1 week (Japanese cohort: 100% [95% CI, 97.5–100.0]; PROGNOSIS validation cohort: 99.3% [95% CI, 97.9–99.9]), and the PPV for ruling in preeclampsia within 4 weeks (32.4% versus 36.7%) and the area under the ROC curve values for the combined endpoint were similar for the PROGNOSIS Asia Japan cohort versus PROGNOSIS (1 week: 94.2% versus 88.3%; 4 weeks: 85.9% versus 86.4%), despite some differences between cohorts. For each of these measures, the 95% confidence intervals overlapped between cohorts.

Our findings are also consistent with the results of the randomized INSPIRE study, which recruited pregnant women (≥18 years; gestational weeks 24–37) with a clinical suspicion of preeclampsia at a single tertiary referral center in the United Kingdom [25]. The NPV for ruling out preeclampsia within 1 week was 100% in the present Japanese cohort versus 100% in INSPIRE (with standard clinical management plus sFlt-1/PlGF ratio; 99.2% for the ratio only) [25].

The findings of the present study are applicable to Japanese women presenting with clinically suspected preeclampsia, adding to growing evidence around the predictive value of the sFlt-1/PlGF ratio (cutoff 38) in Caucasian and Asian women [19, 20]. The varied baseline characteristics between the validation cohorts of PROGNOSIS Asia (including this subanalysis for Japan) and that of Zeisler et al. [19] provide support for universal use of the cutoff value of 38 for the Elecsys sFlt-1/PlGF immunoassay ratio, irrespective of individual characteristics. The PROGNOSIS Asia and PROGNOSIS studies both used the Elecsys sFlt-1 and PlGF immunoassays to determine the sFlt-1/PlGF ratio. Importantly, the optimal sFlt-1/PlGF ratio cutoff to predict preeclampsia may differ with immunoassays from other manufacturers. For example, it has been shown that the cutoffs used for the Elecsys sFlt-1/PlGF ratio are not transferrable to the Brahms Kryptor sFlt-1/PlGF immunoassay [26, 27].

The strengths of this work include analyses based on a well-defined sample cohort recruited from multiple sites across Japan and the use of fully automated immunoassays to derive the sFlt-1/PlGF ratio. Limitations include that the PROGNOSIS Asia study was powered for the primary analysis rather than for the present exploratory subanalysis and that only one MAO occurred, preventing separate evaluation of the predictive performance of the sFlt-1/PlGF ratio for MAOs. Additionally, PROGNOSIS and PROGNOSIS Asia were observational studies. Therefore, randomized trials, such as the INSPIRE study [25], are warranted to compare standard of care versus sFlt-1/PlGF-guided prediction for reducing hospitalizations and improving clinical outcomes in women presenting with clinical suspicion of preeclampsia.

## Conclusions

This subanalysis of Japanese women with suspicion of preeclampsia enrolled in PROGNOSIS Asia showed the high predictive value of the Elecsys sFlt-1/PlGF ratio cutoff of 38 for short-term prediction of preeclampsia in this population, supporting its use alongside other diagnostic and clinical information. Adoption of the sFlt-1/PlGF ratio into clinical practice has the potential to improve both outcomes for pregnant women with suspected preeclampsia and fetal outcomes.

## Supplementary information

Supplementary Fig. 1

Supplementary Fig. 2

Supplementary Fig. 3

## Data Availability

The data that support the findings of this study are available from Roche Diagnostics Ltd but restrictions apply to the availability of these data, which were used under license for the current study, and therefore, are not publicly available. Data are, however, available from the authors upon reasonable request and with permission of Roche Diagnostics Ltd.
