# Supplementary material for: Short-term prediction of preeclampsia using the sFlt-1/PlGF ratio: a subanalysis of pregnant Japanese women from the PROGNOSIS Asia study
Source: Hypertens Res. 2021 Mar 17;44(7):813–21. doi: 10.1038/s41440-021-00629-x (PMC8255209; doi:10.1038/s41440-021-00629-x)
Supplement: Supplementary file 1 — Supplementary Fig. 1 [file 41440_2021_629_MOESM1_ESM.docx]

**Supplementary Fig. 1.** Performance of the sFlt-1/PlGF ratio for predicting FAOs within 1 week and within 4 weeks.^a^ **Panel A** shows the distribution of sFlt-1/PlGF ratios at baseline for participants who developed or did not develop ≥1 FAO within 1 week and within 4 weeks.^b^ **Panel B** shows the performance of the ratio for predicting an FAO within 4 weeks in all participants and by preeclampsia status within 4 weeks.

^a^176 participants from Japan were eligible for this analysis.

^b^ Boxes represent the median and interquartile range; the lower whisker represents the larger of the minimum ratios and the 25^th^ quartile to 1.5x interquartile range, whilst the higher whisker represents the smaller of the maximum ratios and the 75^th^ quartile to 1.5x interquartile range, in log-scale.

FAO, foetal adverse outcome; PE, preeclampsia; PlGF, placental growth factor; sFlt-1, soluble fms-like tyrosine kinase 1.


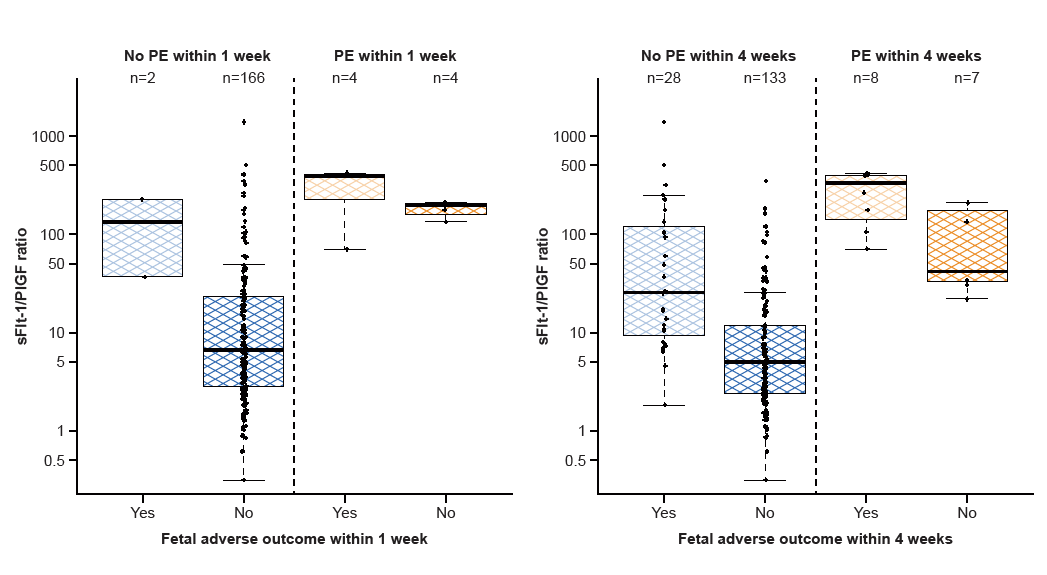
**A**


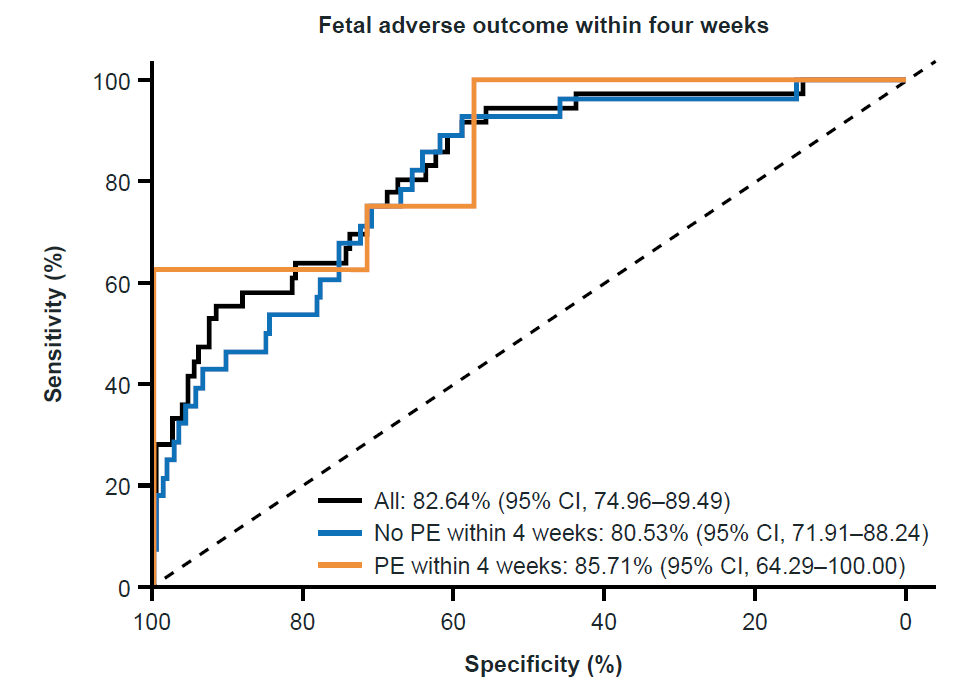
**B**
