# Supplementary material for: Short-term prediction of preeclampsia using the sFlt-1/PlGF ratio: a subanalysis of pregnant Japanese women from the PROGNOSIS Asia study
Source: Hypertens Res. 2021 Mar 17;44(7):813–21. doi: 10.1038/s41440-021-00629-x (PMC8255209; doi:10.1038/s41440-021-00629-x)

**Supplementary Fig. 2.** Distribution of complete pregnancy duration according to sFlt-1/PlGF ratio and by preeclampsia status at baseline visit.^a,b^

^a^179 participants from Japan were eligible for this analysis.

^b^Boxes represent the median and interquartile range; the lower whisker represents the larger of the minimum ratios and the 25^th^ quartile to 1.5x interquartile range, whilst the higher whisker represents the smaller of the maximum ratios and the 75^th^ quartile to 1.5x interquartile range.

PE, preeclampsia; PlGF, placental growth factor; sFlt-1, soluble fms-like tyrosine kinase 1.


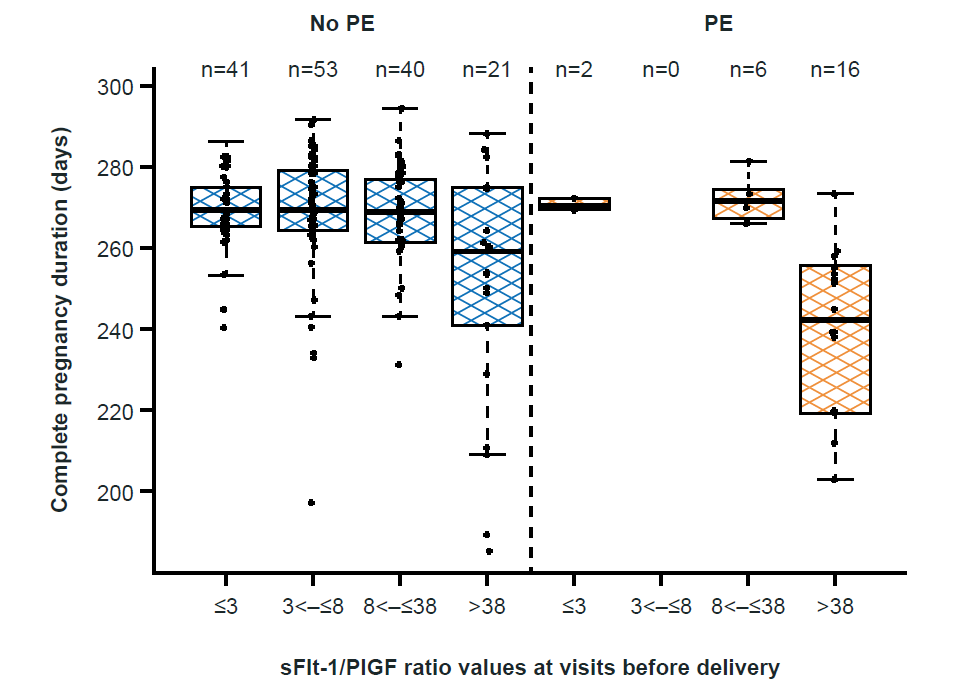

Supplement: Supplementary file 2 — Supplementary Fig. 2 [file 41440_2021_629_MOESM2_ESM.docx]
