# Supplementary material for: Short-term prediction of preeclampsia using the sFlt-1/PlGF ratio: a subanalysis of pregnant Japanese women from the PROGNOSIS Asia study
Source: Hypertens Res. 2021 Mar 17;44(7):813–21. doi: 10.1038/s41440-021-00629-x (PMC8255209; doi:10.1038/s41440-021-00629-x)

**Supplementary Fig. 3**. Baseline sFlt-1/PlGF ratio in women who did not develop preeclampsia, by delivery type.^a,b^

^a^139 participants from Japan were eligible for this analysis.

^b^Boxes represent the median and interquartile range; the lower whisker represents the larger of the minimum ratios and the 25^th^ quartile to 1.5x interquartile range, whilst the higher whisker represents the smaller of the maximum ratios and the 75^th^ quartile to 1.5x interquartile range.

PlGF, placental growth factor; sFlt-1, soluble fms-like tyrosine kinase 1.


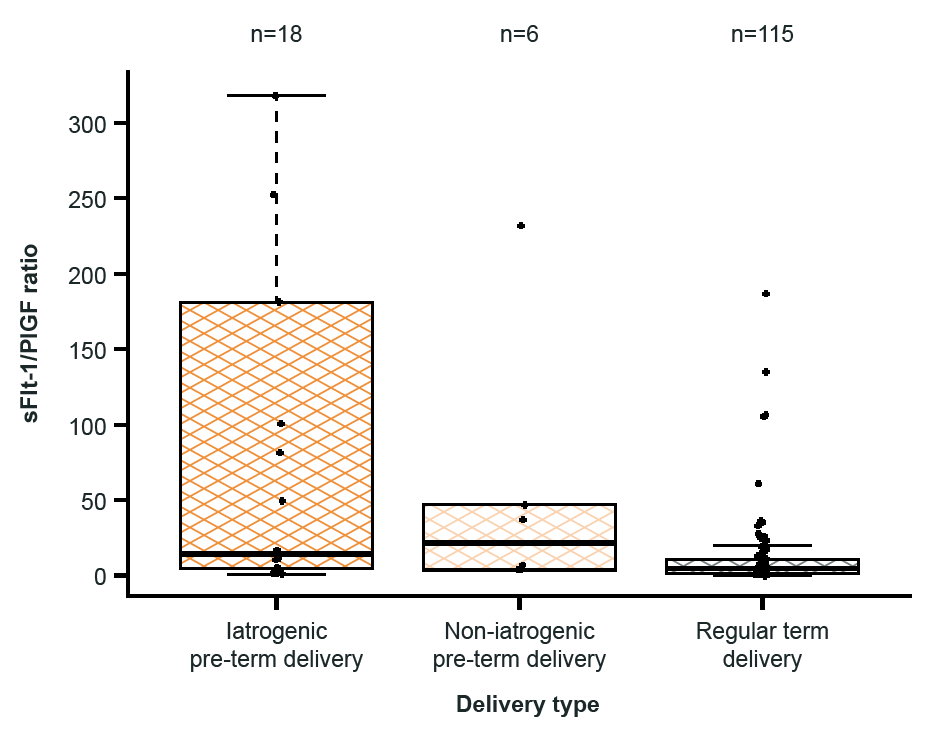

Supplement: Supplementary file 3 — Supplementary Fig. 3 [file 41440_2021_629_MOESM3_ESM.docx]
